# Increased memory confidence and delusions in Alzheimer’s disease: a preliminary study

**DOI:** 10.1080/13554794.2024.2426267

**Published:** 2024-11-08

**Authors:** Emma McLachlan, Kathy Liu, Lauren Huzzey, Neil Burgess, Suzanne Reeves, Robert Howard

**Affiliations:** a Division of Psychiatry, University College London, London, UK; b Enfield Memory Service, Barnet, Enfield and Haringey NHS Mental Health Trust, London, UK; c UCL Institute of Cognitive Neuroscience and UCL Queen Square Institute of Neurology, University College London, London, UK; d Wellcome Centre for Human Neuroimaging, University College London, London, UK

**Keywords:** Alzheimer’s disease, delusions, psychosis, metamemory, Confidence

## Abstract

There is uncertainty about whether delusion formation in Alzheimer’s disease (AD) can be explained by false memories. “Metamemory,” the ability to self-evaluate memory and identify memory errors, is impaired in people with delusions in schizophrenia. Our objective was to investigate whether false memory and metamemory were associated with delusions in AD. Participants with mild AD, with and without delusions, completed a computerized word recognition task and a metamemory measure. Group differences were compared using independent-samples t-tests or Mann Whitney tests. Significant findings were explored through binary logistic regression modeling. Participants with delusions (*n* = 10) gave more high confidence responses, significantly so for correct responses; percentage of high confidence correct responses for those with delusions (mean (SD)) was 69.7% (31.0%) and for those without (*n* = 14) was 43.5% (29.9%); *t*_22_ = -2.09, *p* = .049. This remained significant when sex was included in regression modeling; for each 1.0% increase in high confidence correct responses, participants were 5.4% more likely to have delusions (Exp(β) 1.054, 95% CI 1.007–1.105, *p* = .025). Findings provide tentative support for a link between metamemory and delusions in AD. This should be explored in a larger sample as it has potential implications for treatment.

## Introduction

Individuals with Alzheimer’s disease (AD) are susceptible to both delusions (fixed false beliefs relating to the present, occurring in up to 50% of people with AD) and false memories (fixed memory-related false beliefs relating to the past, occurring in up to 90% of people with AD) (Ropacki & Jeste, 2005; Turk et al., 2020). The Deese-Roediger-McDermott (DRM) paradigm is a widely used index of false memory, which requires participants to distinguish previously presented words (for example, medicine, sick, nurse) from highly related but not previously presented “lure” words (for example, doctor) and unrelated and not previously presented words (Roediger & McDermott, 1995). In health, DRM false memories correlate with sub-clinical delusional ideation (Dehon et al., 2008). However, for individuals with schizophrenia evidence is mixed; some studies using the DRM have found an increase in false memories, particularly in those with current psychosis symptoms (Bhatt et al., 2010), while others have not found any increase in false memories (Huron & Danion, 2002). There is limited data on the relationship between false memories and delusions in AD.

“Metamemory” is the ability to self-evaluate memory capabilities and includes the ability to recognize and correct false memories. Impaired task-specific metamemory has been proposed as a mechanism for delusion formation in schizophrenia (Moritz et al., 2005). Patients with schizophrenia have higher confidence in memory errors than healthy controls, and those who are currently experiencing delusions have higher confidence in memory errors than those who are not (Moritz & Woodward, 2002). Delusions in AD are associated with global metamemory impairment (Migliorelli et al., 1995), but no previous studies have explored how task-specific metamemory relates to delusions in AD.

### Objectives

The aim of this study was to investigate the relationship between false memory, metamemory and delusions in AD.

We hypothesized that impaired metamemory may function as a “first factor” neuropsychological impairment leading to delusion formation (Coltheart, 2010), and that individuals with delusions in AD would have higher confidence in memory errors than those without delusions in AD.

## Materials and methods

### Sample

Participants with mild AD (McKhann et al. (1984); sMMSE scores ≥ 22; age > 55) were recruited from local memory services and from the Join Dementia Research register (www.joindementiaresearch.nihr.ac.uk). Additional inclusion criteria were that participants were required to have the capacity to provide informed consent and be fluent in English, as translated versions of the tasks were not available. Exclusion criteria were current or past history of major psychiatric or neurological illness; current or past alcohol or drug misuse; presence of parkinsonian symptoms suggestive of Lewy body dementia (score >8 on the modified Unified Parkinson’s Disease Rating Scale [UPDRS]; Ballard et al.,^1997^); insufficient visual or auditory acuity to complete the task. The caregiver-rated Neuropsychiatric Inventory (NPI) was completed to screen for presence of delusions. The study was approved by the University College London and University College London Hospital Joint Research Office, Westminster Research Ethics Committee and the Health Research Authority.

### False memory and metamemory measures

A computerized version of the DRM was used. Participants were shown 15 lists of four words, selected at random from 40 DRM lists of semantic associates (Roediger & McDermott, 1995). These were the four words most closely associated with the “lure” word for each list, displayed sequentially on screen for 3000 ms, followed by a 1000 ms mask. Within each list words were presented in order of increasing association with the “lure” word, which is not shown (for example, eye, sewing, sharp, haystack; “lure” word needle). Participants read the words aloud and were aware that they would subsequently have their recall tested.

Participants then completed a recognition task in which 75 words appeared on screen sequentially. This included 15 “lure” words, the two words most closely associated with the “lure” from each of the 15 lists and 30 unrelated words with no semantic association to the DRM word lists (Chadwick et al., 2016). After participants answered whether each word was “old” or “new” they rated their confidence in their answer on a three-point scale (complete guess – fairly confident − 100% confident). Discrimination (d’) and response bias (c) were calculated for recognition memory performance as in Macmillan (1993).

### Statistical analyses

Analyses were carried out in SPSS, with results considered significant at *p* < .05. Demographic characteristics and task performance were compared between the control and delusion groups using independent-samples t-tests or Mann Whitney tests if assumptions of parametric data were violated, and chi-squared tests for categorical variables. Where there was a significant difference in test measure, comparisons were re-run excluding outliers. Significant findings were further explored using binary logistic regression, with delusion group as the dependent variable. Given the relatively small sample size, potential confounding variables were identified a priori: age, sex, years of education, sMMSE score, cholinesterase inhibitor prescription and category fluency on ACE-III. Univariable binary logistic regression analyses were carried out for test measures with a significant between-group difference and each potential confounding variable. Confounding variables that reached the more liberal threshold of *p* < .10 were then included in the full multivariable binary logistic regression model, predicting presence of delusions. Further model diagnostics were run for models with significant results (VIF scores, Box-Tidwell test and Hosmer and Lemeshow test).

## Results

### Sample characteristics

Of the 24 participants (10 with delusions, 14 without delusions), 15 (62.5%) were male and 9 (37.5%) were female; mean (SD) age 83.7 (6.7) years, sMMSE score 25.6 (2.3), ACE-III score 75.7 (14.9), years of education 14.5 (4.5). There were no statistically significant differences between the groups in age, sex, race, years of education, sMMSE or ACE-III score, or proportion prescribed cognitive enhancers or antidepressants. No participants were prescribed other psychotropic medications. Scores on GDS-15 and UPDRS were below cutoffs for exclusion from the study. The mean (SD) NPI score for the delusion group was 13.2 (12.2), with all participants currently experiencing delusion symptoms. Two of the 10 participants in the delusion group were also experiencing hallucinations.

### False memory and metamemory performance in participants with delusions vs control participants

**Figure 1.**
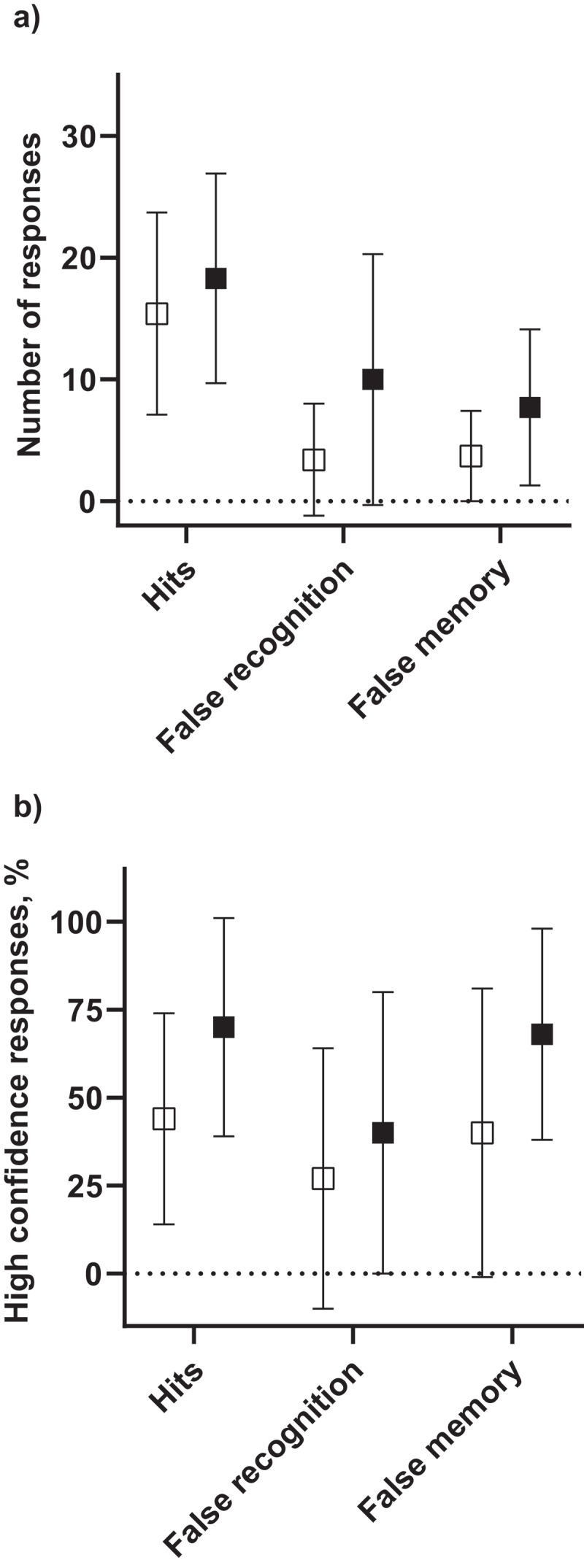
a). Mean (SD) number of DRM responses by response type in those with delusions vs the control group. b). Mean (SD) percentage of high confidence responses by response type in those with delusions vs the control group.

There was a trend toward those with delusions having greater hit, false recognition and false memory rates, associated with reduced discrimination and response bias, however none of these differences reached statistical significance. Those with delusions were more confident in their responses across all response types, a finding that reached statistical significance for correct responses (Table 1, Figure 1).

Empty squares = control group, black squares = delusion group

DRM = Deese-Roediger-McDermott paradigm

**Table 1.** Performance on the DRM and metamemory task for those with delusions vs the control group.

Task component	Control (*n* = 14)	Delusions (*n* = 10)	Control vs Delusions, *P* Value
Hits, mean (SD) (Maximum total 30)	15.4 (8.3)	18.3 (8.6)	*t*_22_ = −.823, *p* = .420
False recognition, median (IQR) (Maximum total 30)	1.5 (0 – 5)	7.5 (0.8 – 18.8)	*U* = 45.0, *p* = .154
False memories, median (IQR) (Maximum total 15)	2 (1 – 5.8)	9 (0 – 13.5)	*U* = 50.0, *p* = .259
Discrimination (d’), mean (SD)	1.5 (0.8)	1.0 (0.8)	*t*_22_ = 1.506, *p* = .146
Response bias (c), mean (SD)	0.7 (0.7)	0.1 (1.1)	*t*_22_ = 1.538, *p* = .138
High confidence hits, mean (SD), %	43.5 (29.9)	69.7 (31.0)	*t*_22_ = −2.085, ***p*** **= .049**
High confidence false recognition^†^, median (IQR), %	5.6 (0.0 – 56.3)	40.4 (0.0 – 74.2)	*U* = 33.0, *p* = .573
High confidence false memories^‡^, median (IQR), %	25.0 (0.0 – 80.0)	61.5 (33.3 – 100.0)	*U* = 26.0, *p* = .135

^†^Control group *n* = 10, delusion group *n* = 8 for false recognition for false memory confidence responses.

^‡^Control group *n* = 13, delusion group *n* = 7 for false memory confidence responses.

DRM = Deese-Roediger-McDermott paradigm.

The univariable model including percentage of high confidence hits was significant (*p* = .037). Sex was the only potential covariate that had *p* < .10 and was therefore the only covariate included for multivariable regression analyses. The model including sex was significant (*X*^2^ (2, *n* = 24) = 11.656, *p* = .003). For each 1% increase in high confidence hits, participants were 5.4% more likely to be in the delusion group (Exp(B) 1.054, 95% CI 1.007–1.105, *p* = .025). Model diagnostics indicated good model fit.

With one identified outlier excluded, percentage of high confidence correct responses remained greater in those with delusions (*n* = 9) compared to the control group (*n* = 14), but no longer reached statistical significance (67.1% (31.7%) compared to 43.5% (29.9%); *t*_21_ = −1.806, *p* = .085). However, percentage of high confidence hits remained significant in the regression model including sex, with participants 5.0% more likely to be in the delusion group for each 1% increase in high confidence hits with this outlier excluded (Exp(β) 1.050, 95% CI 1.003–1.099, *p* = .039).

## Discussion

In this small sample of people with AD, there was evidence that individuals with delusions had increased confidence in all memory responses compared to those without delusions, a finding that reached statistical significance for correct responses. High confidence responses remained significantly associated with delusions when sex was included in multivariable binary logistic regression modeling. This is similar to findings of Evans et al. (2019) that individuals with delusion-like ideation are more highly confident in correct responses, and Moritz and Woodward (2002) that presence of delusions in schizophrenia correlates with increased confidence for correct responses. However, this is not a consistent finding, with Bhatt et al. (2010) finding no overall difference in high confidence false recognition between those with and without delusions in schizophrenia. While it did not reach statistical significance, the trend toward increased confidence in false recognition and false memory is similar to findings in schizophrenia research: individuals with schizophrenia are more highly confident in memory errors than controls (Bhatt et al., 2010; Moritz & Woodward, 2002). Of note, confidence rating scales used to assess metamemory vary widely between studies, from binary judgments to Likert scales with various different wordings and continuous measures, for example, length of a button press. This may go some way to explaining a lack of consistency in findings. Given that those with delusions in AD are also found to have increased anosognosia scores compared to those without (Migliorelli et al., 1995), it would be valuable to include a measure of disease insight in future studies.

### Limitations

This study has several limitations, of which perhaps the most significant is the small sample size. Despite this, trends can be observed in the data and one finding reached statistical significance. We also took an approach to regression modeling which limited the number of covariates, aiming to reduce the risk of overfitting.

Other limitations are shared by all studies in this participant group, including the challenges of accurately diagnosing both AD and delusions. While UPDRS scores were below cut off, given presence of hallucinations in two participants, it cannot be completely ruled out that these individuals had undiagnosed Lewy body dementia.

## Conclusions

Findings provide tentative support for a link between metamemory and delusions in AD. A 10 week course of metamemory training has been found to improve cognitive performance in older adults with subjective memory complaints, with improvements correlating with increased prefrontal cortical thickness (Youn et al., 2019). The findings of the current study are in a cohort with relatively mild AD, who are therefore likely to be able to engage with such low-cost low-risk cognitive interventions. As such, our finding is worthy of further exploration in a larger sample, given the potential implications for management of delusions.

## Data Availability

The participants of this study did not give written consent for their data to be shared publicly.
